# Association between personality traits and glycemic control after inpatient diabetes education

**DOI:** 10.1016/j.metop.2023.100244

**Published:** 2023-04-14

**Authors:** Taisuke Uchida, Hiroaki Ueno, Ayaka Konagata, Takayuki Nakamura, Norifumi Taniguchi, Hiroki Nabekura, Fumiko Kogo, Yuma Nagatomo, Yuri Tanaka, Koichiro Shimizu, Tomomi Shiiya, Hideki Yamaguchi, Kazuya Shimoda

**Affiliations:** a Division of Hematology, Diabetes, and Endocrinology, Department of Internal Medicine, Faculty of Medicine, University of Miyazaki, 5200 Kihara, Kiyotake, Miyazaki, 889-1692, Japan; b Koga General Hospital, 1749-1 Sudaki, Ikeuchi, Miyazaki, 880-0041, Japan

**Keywords:** Diabetes, Diabetes education, Diabetes self-management education and support, Personality trait

## Abstract

**Aims:**

The longitudinal effect of personality traits on glycemic control is unclear. This prospective observational study explored the relationship between personality traits and glycemic control in patients with uncontrolled diabetes after inpatient diabetes education.

**Methods:**

Patients with diabetes mellitus (HbA1c ≥ 7.5%, measured by high-performance liquid chromatography) who received inpatient diabetes education were scored on the Big Five personality traits: neuroticism, extraversion, openness, agreeableness, and conscientiousness. Multiple linear analysis was used to determine whether any personality traits were independently associated with HbA1c on admission and HbA1c change from admission to 1, 3, and 6 months after discharge.

**Results:**

One hundred seventeen participants (mean age 60.4 ± 14.5 years; 59.0% male) were enrolled. HbA1c values on admission and 1, 3, and 6 months after discharge were 10.2 ± 2.1%, 8.3 ± 1.4%, 7.6 ± 1.4%, and 7.7 ± 1.5%, respectively. Multiple linear analysis showed that no personality traits were associated with HbA1c on admission. Neuroticism was negatively associated with the HbA1c change from admission to 3 months (β = −0.192, *P* = 0.025) and 6 months after discharge (β = −0.164, *P* = 0.043).

**Conclusions:**

Neuroticism was associated with good long-term glycemic control after inpatient diabetes education.

## Highlights

• The longitudinal effects of personality traits on glycemic control are unclear.
• We investigated the influences of Big Five personality traits on glycemic control.
• The high-neuroticism group had the greatest reduction in HbA1c after DM education.
• Neuroticism was independently associated with long-term glycemic control.

## 1 Introduction

Inpatient diabetes self-management education and support (DSMES) improves glycemic control after discharge, but some patients experience worsening of glycemic control and readmission [1,2]. Regarding mental disorders, depression and psychosis were associated with readmission after inpatient DSMES [3]. Symptoms of mental disorders are known to lie on a continuum with normal personality traits [4]. The Big Five personality model includes five traits, namely neuroticism, extraversion, openness, agreeableness, and conscientiousness, and has been widely used in clinical research to conceptualize personality traits [5]. There have been several reports on the association between personality traits and glycemic control in patients with diabetes, but the conclusions are controversial [[6], [7], [8], [9], [10], [11]]. A previous longitudinal study [6] examined this association after outpatient DSMES in patients with relatively mild diabetes, but no studies have assessed it after inpatient DSMES in patients with severe diabetes. This study investigated the association between post-discharge glycemic control and personality traits in patients with uncontrolled diabetes who underwent inpatient DSMES.

## 2 Material and methods

### 2.1 Study design

This study was approved by the ethics board of the University of Miyazaki (#O-0834). The main outcome was the association between personality traits and glycemic control after inpatient diabetes education. Patients recruited for this study who had uncontrolled diabetes requiring treatment modification and diabetes education, including that related to enhanced diet and exercise therapy, and who underwent inpatient DSMES from January 2021 to December 2021. All patients had received diet and/or drug therapy for at least 6 months, and their diabetes status was verified by a clinician based on glycated hemoglobin (HbA1c) levels. Patients with HbA1c ≥ 7.5% were eligible for the analysis. HbA1c, expressed as the National Glycohemoglobin Standardization Program (NGSP) value [12], was measured by high-performance liquid chromatography (Tosoh Co., Tokyo, Japan). Written informed consent was obtained from all patients. Patients younger than 20 years with psychiatric disorders or moderate to severe cognitive impairment were excluded. Physical and laboratory findings and treatments at admission and at 1, 3, and 6 months after discharge were evaluated. Patients treated at other hospitals after discharge were asked to provide information from those hospitals. A total of 117 patients who completed the longitudinal analysis were included in the study.

### 2.2 Personality traits

The Japanese version of the Ten-Item Personality Inventory (TIPI-J) was used to classify personality traits. Ten questions, each scored from 1 to 7, were used to score five traits: neuroticism, extraversion, openness, agreeableness, and conscientiousness [13,14]. Participants completed the TIPI-J in a self-assessment format during their hospitalization. Participants were divided into high- and low-score groups for each personality trait, defined relative to the median TIPI-J scores in this study.

### 2.3 Inpatient DSMES

As part of the inpatient DSMES, patients received diabetes-related education from an endocrinologist, specially trained nurse, pharmacist, and registered dietitian. In addition, patients could view health information provided on the hospital's closed-circuit TV channel. Each patient's insulin regimen, oral hypoglycemic medications (OHA), and diet were adjusted during hospitalization.

### 2.4 Statistics analysis

**Fig. 1:**
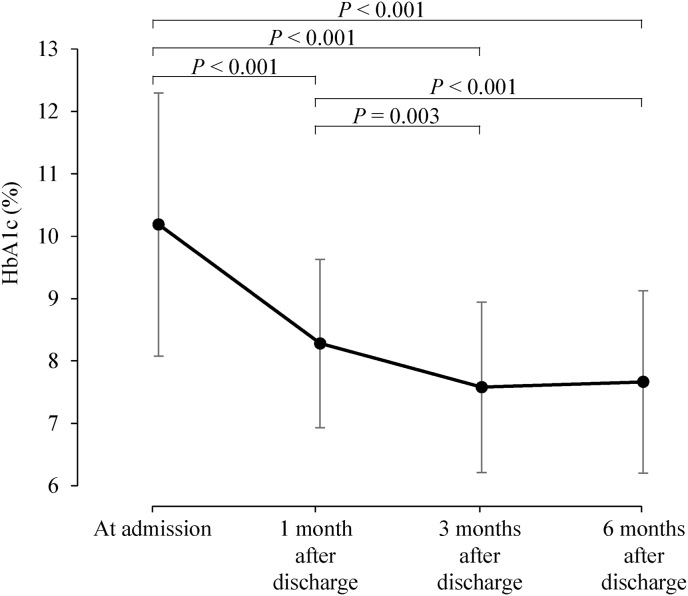
Prospective measurement of HbA1c at admission and after discharge.

**Fig. 2:**
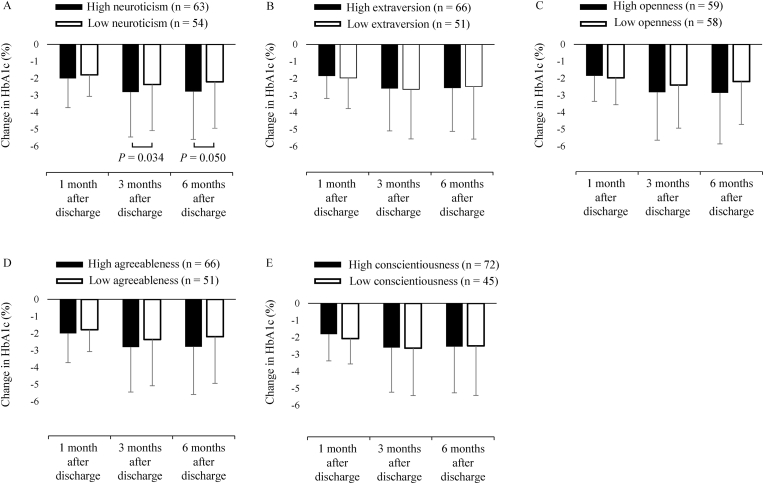
HbA1c change in groups with high and low personality scores after discharge.

Continuous variables were analyzed using post-hoc analysis with Bonferroni's test (Fig. 1, Fig. 2). Simple and multiple regression analyses were conducted to detect factors independently associated with glycemic control (HbA1c on admission and HbA1c change from admission to 1, 3, and 6 months after discharge), sex, age, type of diabetes, alcohol habits, smoking habits, exercise habits, daily injection frequency, daily oral administration (OA) frequency, body mass index (BMI), and personality traits. For linear analysis, we defined categorical variables as follows: female = 0, male = 1; type 1 diabetes = 0, type 2 diabetes = 1; absence of habitual alcohol consumption = 0, presence of habitual alcohol consumption = 1; absence of smoking = 0, presence of smoking = 1; no exercise = 0, exercise once or twice a week = 1, exercise three or four times a week = 2, exercise five or six times a week = 3, exercise every day = 4. “Injection” included insulin injections and glucagon-like peptide-1 receptor agonist (GLP1-RA) injections. R version 3.6.3 was used for all analyses. *P* < 0.05 was accepted as statistically significant.

## 3 Results

Patients’ baseline characteristics on admission (n = 117) are shown in Table 1. The mean age was 60.4 ± 14.5 years (means ± standard deviation, range from 20 to 87 years). The mean duration of diabetes was 11.4 ± 10.5 years (range from 0.5 to 43 years). Inpatient DSMES was associated with significantly lower HbA1c at 1, 3, and 6 months after discharge than at admission (*P* values vs admission; <0.001, <0.001, and <0.001, respectively, Fig. 1), and HbA1c was not significantly different between 3 and 6 months (*P* = 1.000). Compared to the BMI at admission, that at 1 and 3 months after discharge was significantly lower, but there was no significant difference between baseline and 6 months (*P* values vs admission; <0.001, 0.015, and 0.261, respectively, Supplemental Table 1). In terms of diabetic treatment, doses of insulin and GLP-1RA were higher after admission (Supplemental Table 1). Regarding OHAs, doses of biguanides and sodium-glucose cotransporter-2 inhibitors were higher after admission, while those of alpha-glucosidase inhibitors were lower (Supplemental Table 1).

**Table 1:** Clinical characteristics of subjects.

	Variable
Number of patients	117
Sex; male (%)	69 (59.0)
Age (years)	60.4 ± 14.5
BMI (kg/m^2^)	24.9 ± 5.1
Type of diabetes; T2DM (%)	97 (82.9)
Duration (years)	11.4 ± 10.5
Alcohol; none (%)	57 (48.7)
Smoking; none (%)	86 (73.5)
Exercise (%)	
0	77 (65.8)
1	8 (6.8)
2	4 (3.4)
3	9 (7.7)
4	19 (16.2)
Daily injection frequency	1.3 ± 1.6
Daily oral administration frequency	2.7 ± 1.7
HbA1c (%)	
at admission	10.2 ± 2.1
1 month after discharge	8.3 ± 1.4
3 months after discharge	7.6 ± 1.4
6 months after discharge	7.7 ± 1.5
Personality traits	
Neuroticism	3.9 ± 1.4
Extraversion	4.0 ± 1.4
Openness	3.9 ± 1.0
Agreeableness	5.3 ± 1.0
Conscientiousness	3.8 ± 1.3

T2DM, type 2 diabetes mellitus; BMI, body mass index.

Values are expressed as mean ± standard deviation or percentage.

In single regression analysis, extraversion was negatively correlated with HbA1c on admission (Table 2). In multiple regression analysis, HbA1c on admission was independently associated with sex, age, type of diabetes, and exercise habits, but there was no significant association with personality traits (Table 2). In terms of HbA1c change after discharge, there was no significant correlation between any personality trait and HbA1c change 1 month after discharge (Table 3). Neuroticism was independently and negatively associated with HbA1c change at 3 months (β = −0.192, *P* = 0.025) and 6 months after discharge (β = −0.164, *P* = 0.043) (Table 3).

**Table 2:** Simple and multiple linear regression analyses of factors associated with HbA1c on admission.

	r	*P*	β	*P*
Sex	0.594	0.136	**−0.183**	**0.019**
Age	−0.171	0.065	**−0.342**	**0.004**
Type of diabetes	0.842	0.106	−**0.170**	**0.025**
Alcohol	0.047	0.614	0.066	0.502
Smoking	−0.048	0.607	0.124	0.211
Exercise	**−0.209**	**0.024**	**−0.276**	**0.005**
Injection frequency	−0.143	0.125	−0.010	0.926
OA frequency	−0.077	0.411	−0.148	0.146
BMI	−0.025	0.514	**−0.280**	**0.007**
Personality traits				
Neuroticism	0.043	0.645	0.058	0.546
Extraversion	**−0.196**	**0.034**	−0.117	0.262
Openness	−0.016	0.862	−0.112	0.257
Agreeableness	0.037	0.694	0.061	0.517
Conscientiousness	−0.075	0.421	−0.024	0.811
Adjusted R-squared			0.144	
F-statistic			2.388 (*P* = 0.006)	

OA, oral administration; BMI, body mass index.

*P* < 0.05 in bold is considered significant.

**Table 3:** Multiple linear regression analyses of factors associated with HbA1c change from admission.

HbA1c change (%)	1 month after discharge		3 months after discharge		6 months after discharge	
	−1.9 ± 1.5		−2.6 ± 2.7		−2.5 ± 2.8	
	β	*P*	β	*P*	β	*P*
Sex	0.055	0.177	0.007	0.162	0.049	0.218
Age	0.155	0.117	**0.310**	**<0.001**	**0.330**	**<0.001**
Type of diabetes	**−0.262**	**0.019**	**−0.302**	**0.001**	**−0.324**	**0.001**
Alcohol	**−**0.087	0.383	−0.137	0.127	−0.121	0.175
Smoking	0.165	0.097	**0.245**	**0.007**	**0.209**	**0.019**
Exercise	**0.210**	**0.028**	**0.176**	**0.041**	0.164	0.054
Injection frequency	0.048	0.656	0.123	0.205	0.130	0.178
OA frequency	**0.236**	**0.026**	**0.237**	**0.013**	**0.266**	**0.005**
BMI	**−0.208**	**0.036**	**−0.211**	**0.018**	**−0.201**	**0.023**
Personality traits						
Neuroticism	**−**0.166	0.982	**−0.192**	**0.025**	**−0.164**	**0.043**
Extraversion	0.022	0.820	0.040	0.637	0.022	0.790
Openness	0.136	0.160	0.058	0.502	0.028	0.741
Agreeableness	**−**0.088	0.365	−0.079	0.361	−0.091	0.295
Conscientiousness	0.037	0.693	−0.040	0.633	−0.044	0.598
Adjusted R-squared	0.126		0.299		0.306	
F-statistic	2.196 (*P* = 0.013)		4.536 (*P* < 0.001)		4.648 (*P* < 0.001)	

OA, oral administration; BMI, body mass index.

*P* < 0.05 in bold is considered significant.

Next, patients were divided into high- and low-score groups for each personality trait (Supplemental Table 2). For all personality traits, the high- and low-score groups showed no significant difference in HbA1c on admission (Supplemental Table 2). Compared to baseline, the high-neuroticism group demonstrated a significantly greater reduction in HbA1c after admission than the low-neuroticism group at 3 months (−3.1 ± 2.9 vs −2.0 ± 2.3%, respectively, *P* = 0.034) and 6 months after discharge (−3.0 ± 3.0 vs −2.0 ± 2.3%, respectively, *P* = 0.050) (Fig. 2).

## 4 Discussion

The present study showed that personality traits, especially neuroticism, were independently associated with long-term glycemic control in Japanese patients with uncontrolled diabetes after inpatient DSMES. This is the first longitudinal observational study of the association between personality traits and glycemic control after inpatient DSMES.

Inpatient DSMES has multiple benefits, including HbA1c reduction, prevention of diabetic microangiopathy and macroangiopathy, and improvement of quality of life and lifestyle behaviors [15]. However, some patients experience subsequent worsening of glycemic control and readmission due to the differences between their home lifestyle and that during hospitalization [1]. Also, 18 patients (15.8%) in our cohort demonstrated higher HbA1c values 6 months after discharge than on admission. Use of insulin injections, diabetic microangiopathy, and psychiatric disorders were associated with worsened glycemic control after discharge [3]. Among psychiatric conditions, depression and anxiety disorders induce apathy and lack of self-care and negatively affect diet and exercise habits, all of which can impair glycemic control [14]. Personality traits are also associated with healthy behaviors such as a good diet, absence of (or minimal) drinking and smoking, and physical activity [[16], [17], [18]]. Furthermore, personality traits are known to significantly affect drug adherence in individuals with chronic disease [19].

However, it has been unclear which personality traits favorably or unfavorably impact glycemic control (Supplemental Table 3) [[6], [7], [8], [9], [10], [11]]. Conflicting data may be due to differences in study design as well as in patient country, age, gender, and religion [20,21]. In our study, HbA1c was not associated with any personality traits on admission. HbA1c change was not associated with any personality traits 1 month after discharge, but was negatively and independently associated with neuroticism 3 and 6 months after discharge. This suggests that short-term glycemic control is improved by inpatient DSMES regardless of personality traits, but long-term glycemic control is influenced by neuroticism. Personality traits are difficult to change without intensive interventions and training [22]. It may be important for patients with diabetes to choose an inpatient or outpatient treatment approach according to their personality traits. In addition, future studies should investigate the tailoring of diabetes treatments to specific personality traits.

## 5 Limitations

This study has several limitations. First, this was a single-center study that included a small number of Japanese patients. However, we were able to compare the longitudinal association between glycemic control and personality traits because the initial observations in all patients began at the time of inpatient DSMES admission. Second, the personality trait assessment in this study did not consider facets and specific traits included in the Big Five personality. Although there are more detailed personality trait assessments, such as the Revised NEO Personality Inventory [23], we used the TIPI-J because it can assess the Big Five personality traits using only 10 questions.

## 6 Conclusion

This present study showed no significant correlation between HbA1c at admission and any of the Big Five personality traits, namely neuroticism, extraversion, openness, agreeableness, and conscientiousness. Following inpatient DSMES, HbA1c was improved at 1 month after discharge but this improvement was not associated with any Big Five personality trait; at 3 and 6 months, only neuroticism was independently associated with good glycemic control. Neuroticism might be the only Big Five personality trait that influences long-term glycemic control after inpatient DSMES for poorly controlled diabetes in Japanese patients. Larger studies are needed to clarify the association between glycemic control and personality traits.

## Disclosure

The authors declare no conflicts of interest.

The protocol for this research project was approved by the ethics board of the University of Miyazaki (Approval No. O-0834), and it conforms to the provisions of the Declaration of Helsinki. This research was registered in the University Hospital Medical Information Network (UMIN, UMIN000047905). Informed consent was obtained from all subjects.

This research is not applicable to animal studies.

## Funding/support

This research was not supported by any grant from funding agencies in the public, commercial, or not-for-profit sectors.

## CRediT authorship contribution statement

**Taisuke Uchida:** Funding acquisition, Conceptualization, Data curation, Formal analysis, Investigation, Methodology, Writing – original draft, All authors have approved the submitted version of the manuscript and have agreed to be accountable for all parts of the work. **Hiroaki Ueno:** Supervision, Validation, Writing – review & editing, All authors have approved the submitted version of the manuscript and have agreed to be accountable for all parts of the work. **Ayaka Konagata:** All authors have approved the submitted version of the manuscript and have agreed to be accountable for all parts of the work. **Takayuki Nakamura:** All authors have approved the submitted version of the manuscript and have agreed to be accountable for all parts of the work. **Norifumi Taniguchi:** Funding acquisition, Writing – review & editing, All authors have approved the submitted version of the manuscript and have agreed to be accountable for all parts of the work. **Hiroki Nabekura:** Funding acquisition, Writing – review & editing, All authors have approved the submitted version of the manuscript and have agreed to be accountable for all parts of the work. **Fumiko Kogo:** Funding acquisition, Writing – review & editing, All authors have approved the submitted version of the manuscript and have agreed to be accountable for all parts of the work. **Yuma Nagatomo:** All authors have approved the submitted version of the manuscript and have agreed to be accountable for all parts of the work. **Yuri Tanaka:** Funding acquisition, Writing – review & editing, All authors have approved the submitted version of the manuscript and have agreed to be accountable for all parts of the work. **Koichiro Shimizu:** Funding acquisition, Writing – review & editing, All authors have approved the submitted version of the manuscript and have agreed to be accountable for all parts of the work. **Tomomi Shiiya:** Funding acquisition, Writing – review & editing, All authors have approved the submitted version of the manuscript and have agreed to be accountable for all parts of the work. **Hideki Yamaguchi:** Supervision, Writing – review & editing, All authors have approved the submitted version of the manuscript and have agreed to be accountable for all parts of the work. **Kazuya Shimoda:** Project administration, Supervision, Validation, Writing – review & editing, All authors have approved the submitted version of the manuscript and have agreed to be accountable for all parts of the work.

## Declaration of competing interest

The authors declare no conflicts of interest.
