# Supplementary material for: Association between personality traits and glycemic control after inpatient diabetes education
Source: Metabol Open. 2023 Apr 14;18:100244. doi: 10.1016/j.metop.2023.100244 (PMC10313504; doi:10.1016/j.metop.2023.100244)
Supplement: Multimedia component 1 [file mmc1.docx]

**Supplemental Table 1.** Chronological changes in patient treatments

|  | At admission | 1 month after discharge | 3 months after discharge | 6 months after discharge |
| --- | --- | --- | --- | --- |
| HbA1c (%) | 10.2 ± 2.1 | **8.3 ± 1.4***** | **7.6 ± 1.4***** | **7.7 ± 1.5***** |
| BMI (kg/m^2^) | 24.9 ± 5.1 | **24.1 ± 4.7***** | **24.4 ± 4.2*** | 24.5 ± 4.3 |
| Daily injection frequency | 1.2 ± 1.6 | **1.8 ± 1.7***** | **1.6 ± 1.6***** | **1.6 ± 1.6***** |
| Number of injections | 0.7 ± 0.9 | **1.1 ± 0.9***** | **1.0 ± 0.9***** | **1.0 ± 0.9***** |
| Long-acting insulin (%) | 49 (41.9) | 71 (60.7) | 70 (59.8) | 66 (56.4) |
| Rapid-acting insulin (%) | 34 (29.1) | 49 (41.9) | 47 (40.2) | 46 (39.3) |
| GLP1RA (injection, %) | 10 (8.5) | **27 (23.1)**** | **26 (22.2)**** | **28 (23.9)***** |
| Insulin dose | 12.7 ± 17.8 | **15.8 ± 17.5*** | 15.2 ± 17.7 | 15.0 ± 17.8 |
| Long-acting insulin (U) | 6.4 ± 9.5 | 8.0 ± 8.9 | 7.7 ± 8.7 | 7.4 ± 8.8 |
| Rapid-acting insulin (U) | 6.3 ± 11.5 | 7.9 ± 11.8 | 7.5 ± 11.6 | 7.6 ± 11.7 |
| Daily OA frequency | 2.7 ± 1.7 | 2.7 ± 1.5 | 2.7 ± 1.4 | 2.7 ± 1.5 |
| Number of OAs | 8.1 ± 7.2 | 8.4 ± 6.3 | 8.4 ± 6.2 | 8.4 ± 6.3 |
| Number of OHAs | 1.4 ± 1.3 | 1.6 ± 1.3 | 1.6 ± 1.2 | 1.6 ± 1.2 |
| Biguanide (%) | 38 (32.5) | **53 (45.3)*** | **55 (47.0)*** | 52 (44.4) |
| DPP4i (%) | 52 (44.4) | 55 (47.0) | 54 (46.2) | 50 (42.7) |
| SGLT2i (%) | 29 (24.8) | **49 (41.9)***** | **50 (42.7)***** | **52 (44.4)***** |
| Alpha-GI (%) | 14 (12.0) | 8 (6.8) | **7 (6.0)*** | **7 (6.0)*** |
| Glinide (%) | 13 (11.1) | 21 (17.9) | 20 (17.1) | 18 (15.4) |
| SU (%) | 7 (6.0) | 2 (1.7) | 2 (1.7) | 2 (1.7) |
| TZD (%) | 6 (5.1) | 0 (0.0) | 0 (0.0) | 0 (0.0) |
| GLP1RA (Oral, %) | 0 (0.0) | 1 (0.9) | 1 (0.9) | 1 (0.9) |

BMI, body mass index; GLP1RA, glucagon-like peptide-1 receptor agonist; OA, oral administration; OHA, oral hypoglycemic agent; DPP4i, dipeptidyl peptidase-4 inhibitor; SGLT2i, sodium-glucose cotransporter-2 inhibitor; Alpha-GI; alpha-glucosidase inhibitor; SU, sulfonylurea; TZD, thiazolidinedione.

Values are expressed as mean ± standard deviation or percentage. *P* < 0.05 in bold is considered significant.

**P* < 0.05 vs admission

***P* < 0.01 vs admission

****P* < 0.001 vs admission

**Supplemental Table 2.** High- vs low-score group for each personality trait

|  | High N | Low N | High E | Low E | High O | Low O | High A | Low A | High C | Low C |
| --- | --- | --- | --- | --- | --- | --- | --- | --- | --- | --- |
| Number of patients | 63 | 54 | 66 | 51 | 59 | 58 | 66 | 51 | 72 | 45 |
| Sex; male (%) | 34 (54.0) | 35 (64.8) | 34 (51.5) | 35 (68.6) | 35 (59.3) | 34 (58.6) | 38 (57.6) | 31 (60.8) | 44 (61.1) | 25 (55.6) |
| Age (years) | 58.0 ± 16.3 | 63.2 ± 11.7 | 59.4 ± 12.9 | 61.7 ± 16.5 | **57.9 ± 14.9** | **63.00 ± 13.8*** | 62.0 ± 14.7 | 58.4 ± 14.1 | 60.9 ± 15.2 | 59.7 ± 13.4 |
| Type of DM; T2DM (%) | 52 (82.5) | 45 (83.3) | 55 (83.3) | 42 (82.4) | 48 (81.4) | 49 (84.5) | 58 (87.9) | 39 (76.5) | 58 (80.6) | 39 (86.7) |
| Duration (years) | 10.9 ± 10.6 | 10.4 ± 12.0 | 10.8 ± 10.1 | 11.9 ± 10.8 | 10.6 ± 10.6 | 12.2 ± 10.4 | 12.4 ± 12.0 | 10.1 ± 7.9 | 10.4 ± 8.0 | 12.0 ± 11.8 |
| Alcohol; none (%) | 36 (57.1) | 21 (38.9) | 30 (45.5) | 27 (52.9) | 35 (59.3) | 25 (43.1) | 39 (59.1) | 21 (41.2) | **28 (38.9)** | **29 (64.4)**** |
| Smoking; none (%) | 48 (76.2) | 38 (70.4) | 45 (68.2) | 41 (80.4) | 43 (72.9) | 43 (74.1) | 47 (71.2) | 39 (76.5) | 50 (69.8) | 36 (80.0) |
| Exercise (%) |  |  |  |  |  |  |  |  |  |  |
| 0 | 40 (63.5) | 55 (67.1) | 41 (62.1) | 36 (70.6) | **38 (64.4)** | **39 (67.2)*** | 43 (65.2) | 34 (66.7) | **44 (61.1)** | **33 (73.3)**** |
| 1 | 5 (7.9) | 3 (5.6) | 6 (9.1) | 2 (3.9) | **1 (1.7)** | **7 (12.1)** | 4 (6.1) | 4 (7.8) | **3 (4.2)** | **5 (11.1)** |
| 2 | 3 (4.8) | 1 (1.9) | 1 (1.5) | 3 (5.9) | **3 (5.1)** | **1 (1.7)** | 2 (3.0) | 2 (3.9) | **1 (1.4)** | **3 (6.7)** |
| 3 | 5 (7.9) | 4 (7.4) | 6 (9.1) | 3 (5.9) | **3 (5.1)** | **6 (10.3)** | 5 (7.6) | 4 (7.8) | **7 (9.7)** | **2 (4.4)** |
| 4 | 10 (15.9) | 9 (16.7) | 12 (18.2) | 7 (13.7) | **14 (23.7)** | **5 (8.6)** | 12 (18.2) | 7 (13.7) | **17 (23.6)** | **2 (4.4)** |
| Injection frequency | 1.0 ± 1.5 | 1.5 ± 1.7 | 1.0 ± 1.5 | 1.6 ± 1.8 | 1.2 ± 1.7 | 1.6 ± 1.3 | **0.9 ± 1.4** | **1.7 ± 1.8** | 1.2 ± 1.6 | 1.2 ± 1.7 |
| OA frequency | 2.7 ± 1.7 | 2.7 ± 1.8 | 2.8 ± 1.7 | 2.7 ± 1.8 | **2.2 ± 1.6** | **3.2 ± 1.8**** | 2.6 ± 1.7 | 2.9 ± 1.8 | 2.6 ± 1.7 | 3.0 ± 1.8 |
| BMI (kg/m^2^) |  |  |  |  |  |  |  |  |  |  |
| At admission | 25.3 ± 5.5 | 24.4 ± 4.5 | 25.3 ± 5.6 | 24.3 ± 4.3 | 24.8 ± 5.4 | 25.0 ± 4.8 | 25.0 ± 5.2 | 24.7 ± 5.0 | **24.1 ± 5.1** | **26.1 ± 4.9*** |
| 1 month after discharge | 24.5 ± 5.1 | 23.7 ± 4.0 | 24.6 ± 5.0 | 23.6 ± 4.2 | 24.0 ± 4.9 | 24.2 ± 4.3 | 24.2 ± 4.6 | 24.0 ± 4.6 | **23.4 ± 4.7** | **25.3 ± 4.4*** |
| 3 months after discharge | 24.6 ± 4.3 | 24.1 ± 3.9 | 24.8 ± 4.5 | 23.9 ± 3.6 | 24.3 ± 4.2 | 24.5 ± 4.1 | 24.2 ± 4.1 | 24.6 ± 4.3 | **23.7 ± 4.1** | **25.5 ± 4.2*** |
| 6 months after discharge | 24.7 ± 4.5 | 24.3 ± 4.0 | 25.1 ± 4.5 | 23.8 ± 3.8 | 24.4 ± 4.3 | 24.6 ± 4.2 | 24.3 ± 4.2 | 24.8 ± 4.3 | **23.8 ± 4.0** | **25.7 ± 4.4*** |
| HbA1c (%) |  |  |  |  |  |  |  |  |  |  |
| At admission | 10.4 ± 2.3 | 10.0 ± 1.9 | 10.0 ± 2.0 | 10.4 ± 2.2 | 10.1 ± 2.4 | 10.2 ± 1.9 | 10.2 ± 2.2 | 10.2 ± 2.0 | 10.0 ± 2.2 | 10.5 ± 2.0 |
| 1 month after discharge | 8.3 ± 1.4 | 8.3 ± 1.3 | 8.1 ± 1.3 | 8.5 ± 1.4 | 8.3 ± 1.4 | 8.3 ± 1.3 | 8.2 ± 1.3 | 8.4 ± 1.5 | 8.2 ± 1.4 | 8.4 ± 1.2 |
| 3 months after discharge | **7.3 ± 1.2** | **7.9 ± 1.5*** | 7.4 ± 1.3 | 7.8 ± 1.5 | **7.3 ± 1.3** | **7.8 ± 1.4*** | 7.4 ± 1.1 | 7.8 ± 1.6 | 7.4 ± 1.3 | 7.9 ± 1.5 |
| 6 months after discharge | **7.4 ± 1.3** | **8.0 ± 1.6*** | 7.4 ± 1.3 | 8.0 ± 1.7 | **7.3 ± 1.4** | **8.1 ± 1.4**** | **7.4 ± 1.2** | **8.0 ± 1.7*** | **7.5 ± 1.5** | **8.0 ± 1.6*** |
| Change in HbA1c from admission |  |  |  |  |  |  |  |  |  |  |
| 1 month after discharge | -2.1 ± 1.7 | -1.7 ± 1.3 | -1.9 ± 1.3 | -2.0 ± 1.8 | -1.9 ± 1.5 | -2.0 ± 1.6 | -2.0 ± 1.6 | -2.0 ± 1.7 | -1.8 ± 1.6 | -2.1 ± 1.5 |
| 3 months after discharge | **-3.1 ± 2.9** | **-2.0 ± 2.3*** | -2.6 ± 2.5 | -2.6 ± 2.9 | -2.4 ± 2.5 | -2.8 ± 2.7 | -2.8 ± 2.7 | -2.4 ± 2.7 | -2.6 ± 2.6 | -2.6 ± 2.8 |
| 6 months after discharge | **-3.0 ± 3.1** | **-2.0 ± 2.3*** | -2.6 ± 2.5 | -2.5 ± 3.1 | -2.9 ± 3.0 | -2.2 ± 2.5 | -2.8 ± 2.8 | -2.2 ± 2.7 | -2.5 ± 2.7 | -2.5 ± 2.9 |
| Personality traits |  |  |  |  |  |  |  |  |  |  |
| Neuroticism | **4.9 ± 0.8** | **3.1 ± 1.0***** | 3.9 ± 1.4 | 3.7 ± 1.4 | 3.9 ± 1.4 | 3.8 ± 1.4 | 3.8 ± 1.4 | 3.9 ± 1.3 | 3.8 ± 1.3 | 4.0 ± 1.5 |
| Extraversion | 4.1 ± 1.3 | 3.9 ± 1.6 | **5.0 ± 0.9** | **2.7 ± 0.6***** | **4.3 ± 1.4** | **3.7 ± 1.4*** | 4.1 ± 1.5 | 3.9 ± 1.4 | 4.2 ± 1.3 | 3.7 ± 1.5 |
| Openness | 3.9 ± 1.0 | 3.9 ± 1.0 | 4.0 ± 1.2 | 3.7 ± 0.8 | **4.7 ± 0.7** | **3.1 ± 0.6***** | 4.0 ± 1.1 | 3.8 ± 0.9 | 3.9 ± 1.1 | 3.8 ± 0.9 |
| Agreeableness | 5.3 ± 1.1 | 5.4 ± 0.9 | 5.4 ± 1.1 | 5.3 ± 1.0 | 5.4 ± 1.1 | 5.3 ± 1.0 | **6.1 ± 0.5** | **4.4 ± 0.7***** | 5.4 ± 1.1 | 5.3 ± 0.9 |
| Conscientiousness | 3.7 ± 1.2 | 4.0 ± 1.4 | **4.1 ± 1.4** | **3.4 ± 1.1**** | 3.9 ± 1.3 | 3.7 ± 1.4 | **4.1 ± 1.3** | **3.5 ± 1.2*** | **4.6 ± 0.9** | **2.5 ± 0.5***** |

N, neuroticism; E, extraversion; O, openness; A, agreeableness; C, conscientiousness, T2DM, type 2 diabetes mellitus; OA, oral administration; BMI, body mass index.

Values are expressed as mean ± standard deviation or percentage. *P* < 0.05 in bold is considered significant.

**P* < 0.05 vs high-score group for each personality trait.

***P* < 0.01 vs high-score group for each personality trait.

****P* < 0.001 vs high-score group for each personality trait.

**Supplemental Table 3.** Associations between personality traits and glycemic control in the current and previous reports

| Study | Participants | Design | Personality measure | Effect on glycemic control | | | Ref |
| --- | --- | --- | --- | --- | --- | --- | --- |
|  |  |  |  | Favorable | No effect | Unfavorable |  |
| Lane  (2000) | N = 105 (56% men, mean age 57 years) including whites (87%), African-Americans (12%), and Asian-Americans (1%) | 12-month longitudinal | NEO-PI-R | Neuroticism | Extraversion, Openness, Conscientiousness | Agreeableness | (6) |
| Skinner  (2014) | N = 1313 (53% men,  mean age 66 years, Australian) | Cross-sectional | BFI44 | (−) | Neuroticism, Extraversion, Openness  Agreeableness, Conscientiousness | (−) | (7) |
| Shim  (2014) | N = 1614 (all female,  mean age 25 years, Korean) | Cross-sectional | NEO-PI-R | Extraversion | Neuroticism, Openness, Agreeableness, Conscientiousness | (−) | (8) |
| Čukić  (2015) | N = 837 (49% male,  mean age 69.6 years, Scottish) | Cross-sectional | NEO-FFI | Openness | Neuroticism, Extraversion, Agreeableness, Conscientiousness | (−) | (9) |
| Esmaeilinasab  (2016) | N = 400 (64% male,  mean age 51 years, Iranian) | Cross-sectional | NEO-PI-R | Extraversion  Conscientiousness | Openness, Agreeableness | Neuroticism | (10) |
| Yasui-Furukori  (2020) | N = 503 (58% male,  mean age 64 years, Japanese) | Cross-sectional | TIPI-J | (−) | Neuroticism, Extraversion, Openness  Agreeableness, Conscientiousness | (−) | (11) |
| This study  (2022) | N = 117 (59% male,  mean age 60 years, Japanese) | 6-month longitudinal | TIPI-J | Neuroticism | Extraversion, Openness, Agreeableness  Conscientiousness | (−) |  |

NEO-PI-R, Revised NEO Personality Inventory; BFI44, 44-item Big Five Personality Inventory; NEO-FFFI, NEO-Five Factor Inventory; TIPI-J, The Japanese version of the Ten-Item Personality Inventory
